# Mechanical circulatory support in cardiogenic shock: a contemporary head-to-head comparison

**DOI:** 10.1007/s10741-026-10612-8

**Published:** 2026-03-24

**Authors:** Stavroula A. Siopi, Polychronis Antonitsis, Georgios T. Karapanagiotidis, Georgios Tagarakis, Christos Voucharas, Kyriakos Anastasiadis

**Affiliations:** Cardiothoracic Department, Aristotle University of Thessaloniki,School of Medicine, AHEPA University Hospital, Thessaloniki, Greece

**Keywords:** Mechanical circulatory support, Cardiogenic shock, Impella, Extracorporeal membrane oxygenation, Intra-aortic balloon pump, Acute heart failure

## Abstract

Modern technology has expanded the armamentarium of temporary mechanical circulatory support devices implemented for hemodynamic stabilization of patients in cardiogenic shock. The predominately used ones are intra-aortic balloon pump (IABP), Impella devices, and extracorporeal life support (ECLS) circuits. They are transcutaneous or surgically implanted, utilized for up to 30 days and can either assist or totally replace cardiac function, providing time for myocardial healing (bridge to recovery) or treatment escalation (bridge to destination therapy, bridge to transplantation). In light of the recently published joint consensus of the Perioperative Quality Initiative and Enhanced Recovery After Surgery Cardiac Society on temporary mechanical circulatory support, purpose of the current review is to provide a succinct head-to-head comparison of widely available devices, as well as current escalation, de-escalation and antithrombotic protocols; thus, it proposes immediately translatable device selection and operation algorithms to medical professionals for optimal patient care and effective management of cardiogenic shock.

**Supplementary Information:**

The online version contains supplementary material available at 10.1007/s10741-026-10612-8.

## Introduction

Cardiogenic shock (CS) is a state of circulatory failure due to cardiac dysfunction, originating from left, right or both ventricles [1]. Patients present with fluid-refractory hypotension and hypoperfusion, leading to end-organ damage. Given the heterogeneity of CS presentations, in 2019 the Society for Coronary Angiography and Interventions (SCAI) established a five-stage classification system (A-E) according to clinical symptoms, biochemical and hemodynamic indices (Online Resource 1) [2]. Most common entities are heart failure-related CS (HFCS), acute myocardial infarction-related CS (AMICS) and post-cardiotomy CS [3, 4]. The initial therapeutic approach includes administration of vasoactive agents. In case of inadequate response, implementation of mechanical circulatory support (MCS), primarily intra-aortic balloon pump (IABP), Impella or extracorporeal life support (ECLS) is recommended. Early MCS initiation is pivotal, as it averts refractory CS and irreversible end-organ dysfunction. This review highlights the key features of each major MCS device, comparatively presents advantages and disadvantages and proposes concrete algorithms pertaining to appropriate device selection, escalation, de-escalation and antithrombotic strategies. Finally, areas of temporary MCS requiring further examination are discussed, suggesting potential future research aims.

## Impella

Impella (Abiomed) is a microaxial catheter-mounted pump which is implanted transcutaneously or surgically to provide temporary left or right ventricular support (Online Resource 2). Left-sided devices are implanted via femoral or axillary arteries and advanced retrograde through the aortic valve, with the inlet in left ventricle (LV) and the outlet in ascending aorta (Online Resource 3) [5]. For right ventricular (RV) support the catheter is implanted through the femoral or internal jugular vein, with the inlet in superior/inferior vena cava and the outlet in pulmonary artery. Impella offers ventricular unloading, reducing end-diastolic pressure (EDP), end-diastolic volume (EDV) and venous congestion, while increasing cardiac output (CO) and mean arterial pressure (MAP). Isovolumic contraction and relaxation periods are lost, resulting in a triangular-shaped pressure-volume loop (PV-loop), shifted leftwards (Online Resource 4). Impella provides a continuous antegrade circulatory flow of 3.7–5.5 l/min for a maximum duration of 14 days [6], though numerous reports of Impella 5.5 implementation for up to 70 days are available [7]. Biventricular support with Impella CP/5.5 as left ventricular assist device (LVAD) and Impella RP/RP Flex as right ventricular assist device (RVAD) is referred to as Bipella and is utilized in cases of biventricular CS [6, 8].

Impella is contraindicated in cases of anatomical constrictions averting proper positioning, valvulopathy of left- or right-sided cardiac valves, implanted mechanical valves, severe peripheral arterial disease and cardiorespiratory failure, while cardiac structural defects should be examined individually, based on their anatomical characteristics and hemodynamic impact [9, 10]. Most frequent complications are acute kidney injury, hemolysis, valvular and vascular injury, ventricular perforation, stroke, insertion-site infection, thrombocytopenia, deep vein thrombosis and arrhythmias [11–13].

Impella has been proven to be an effective temporary LVAD in CS patients in numerous clinical trials (Online Resource 5) [14–17]. Most importantly, the DanGer Shock trial, published in 2024, randomized 355 AMICS patients to either Impella support (*n* = 179) or optimal medical treatment (OMT) (*n* = 176). The trial showed a lower 6-month all-cause mortality in the Impella group (45.8% vs. 58.5%, HR, 0.74; 95% CI, 0.55 to 0.99; *P* = 0.04), with a number-needed-to-treat (NNT) of only 8 patients. These groundbreaking results came at the cost of increased bleeding (BARC 3–5 21.8% vs. 11.9%), need for hemodialysis (41.9% vs. 26.7%) and limb ischemia (5.6% vs. 1.1%), which did not, however, influence overall mortality rates. Survival benefit was sustained up to 10 years despite concomitant adverse events, with lower all-cause mortality rates (52.5% vs. 68.8%, HR 0.70; 95% CI 0.54 to 0.92) and longer median time to death (577 days vs. 61) in Impella group [18]. The results of this trial established Impella as a first-line MCS device in AMICS (Class 2a in American Heart Association guidelines) [14].

## Extracorporeal life support (ECLS)

Extracorporeal life support (ECLS) is increasingly implemented as a temporary MCS in case of cardiorespiratory compromise for up to 30 days [19]. An ECLS circuit consists of a centrifugal continuous-flow pump, a membrane oxygenator, a heater-cooler machine, vascular catheters (cannulae) and circuit tubing. The main configuration providing circulatory support is venoarterial ECLS (VA-ECLS), which consists of a venous-afferent line and an arterial-efferent line usually in femoral vein and artery respectively. VA-ECLS is also indicated in cases of primary isolated RV failure or RV failure secondary to LV failure.

Transfemoral VA-ECLS increases LV afterload due to retrograde blood flow, leading to LV dilatation, reduced stroke volume, increased left atrial (LA) pressure and myocardial oxygen consumption (MVO_2_) and evolution of pulmonary edema (Online Resource 6) [20]. Hence, patients benefit from LV unloading (reduction of LV work) or venting (reduction of LV filling pressures with potential concomitant reduction of LV work). Non-invasive LV unloading is approached with administration of vasodilators, diuresis or hemofiltration [21, 22]. Of great importance is the interplay among perfusion rates, LV afterload and LV filling pressures. While lower LV work is achieved with lower flows (> 1.5 l/min), higher flows approximating 4 l/min are shown to reduce pulmonary-capillary-wedge-pressure (PCWP) given the concomitant reduction in RV preload, thus constituting a non-invasive LV venting method [23]. Invasive LV venting techniques include insertion of a percutaneous catheter in pulmonary artery, LA, or LV or creation of percutaneous atrial septostomy [19]. Contemporary LV unloading options combine IABP or Impella in CS patients under VA-ECLS support (ECPELLA or ECMELLA) [24–26]. As evidenced in a meta-analysis of 5 studies comparing ECLS to ECPELLA, ECPELLA group presented lower all-cause mortality rates (RR: 0.85; 95% CI: 0.75, 0.97; *p* = 0.01), at the cost of increased hemolysis (RR: 1.70; 95% CI: 1.35, 2.15; *p* < 0.00001), while bleeding adverse events were of the same severity and incidence in both groups [24]. In a meta-analysis of 7 studies examining the addition of Impella vs. IABP to VA-ECLS, both groups showed similar short-term all-cause mortality rates [60.8% vs. 64.9%, RR 0.93 (0.71 to 1.21)], while ECPELLA group experienced significantly higher bleeding [57.2% vs. 39.7%, RR 1.66 (1.12 to 2.44)] and hemolysis events [31% vs. 7%, RR 4.61 (1.24 to 17.17)] [27]. In case of post-cardiotomy CS, central VA-ECLS comprising an arterial cannula in distal ascending aorta, a venous cannula in right atrium and a venting cannula in LA or LV can be used, improving hemodynamic profile and shortening support duration [28]. Overt indicators for LV unloading/venting are PCWP > 18 mmHg, pulse pressure < 15 mmHg, LV distension, pulmonary edema, and refractory ventricular arrhythmias [21]. When ECLS supports patients post-LVAD implantation, weaning is performed when circulatory efficiency is restored [mean arterial pressure (MAP) > 65 mmHg, pulse pressure > 10 mmHg, PCWP < 15 mmHg, LV ejection fraction > 30%] and prior to LVAD removal [21].

Most frequent complications during ECLS support are thromboembolism, hemorrhage, vascular injury, insertion-site infection and LV distention [29]. Of great importance is differential hypoxia or Harlequin syndrome, during which fully oxygenated blood is returned to the lower body, while LV ejects poorly oxygenated blood to the upper body, including cerebral and coronary circulation. This is averted with an additional venous efferent line, providing native circulation both antegrade and retrograde with oxygenated blood (V-AV ECLS). Contraindications pertaining to ECLS are non-cardiogenic and irreversible organ failure or heart failure that cannot be treated with LVAD or heart transplantation [30].

The Acute Cardiovascular Care Association and European Society of Cardiology (ESC) recommend the use of VA-ECLS in patients with refractory AMICS or initially classified as SCAI stage D or E [VA-ECLS may be considered for severe CS, in-hospital and out-of-hospital cardiac arrest in individual cases (Class IIb)] [31]. Moreover, under certain conditions (short period in low flows, shockable arrest rhythm, arterial pH > 7.0, low serum lactates, serum creatinine and SOFA score), addition of VA-ECLS during cardiorespiratory resuscitation leads to faster return of spontaneous rhythm with improved neurological outcomes [32, 33]. Trials pertaining to VA-ECLS in CS patients yielded conflicting results, with some proving lower all-cause 30-day mortality and others finding no difference between groups (Online Resource 7) [34–36]. This discrepancy originates from the heterogeneity of inclusion criteria and lack of a universal protocol for VA-ECLS operation. While in ECLS SHOCK and EURO SHOCK trials only AMICS patients were included, ECMO-CS trial stratified patients with CS irrespective of origin. Moreover, the first two studies only included patients with CS onset within 12 h of 24 h respectively, contrary to ECMO-CS that proposed no time-of-onset limitations. ECMO-CS trial did not prove significant difference between VA-ECLS and conservative group, possibly due to lack of stricter inclusion criteria. It should be noted that ECLS SHOCK patients received VA-ECLS support prior to revascularization, whereas EURO SHOCK patients had undergone percutaneous coronary intervention (PCI) prior to CS. Only EURO SHOCK trial proved lower 30-day and 1-year all-cause mortality rates in VA-ECLS group, suggesting that VA-ECLS could potentially benefit AMICS patients post-PCI (successful or attempted). Similarities can be detected in EURO SHOCK and DanGer Shock populations, in terms of CS origin (only included AMICS patients), preceding revascularization, time frames (CS onset within 24 h) and finally in exclusion of individuals with out-of-hospital cardiac arrest, return of spontaneous rhythm (ROSC) and Glasgow-coma-scale < 8 (DanGer Shock) or without ROSC, pH < 7 or delayed cardiopulmonary resuscitation attempts. Both studies showed improved survival in AMICS patients under Impella or VA-ECLS support respectively, highlighting that proper patient selection is crucial for successful interventions.

## Intra-aortic balloon pump (IABP)

Intra-aortic balloon pump (IABP) comprises a self-inflatable balloon positioned on a catheter, which is implanted transcutaneously in descending thoracic aorta. The balloon inflates during diastole and deflates during systole moving blood volume proximally and distally. IABP increases the coronary arterial flow and improves myocardial perfusion. Systemic vascular resistance and systolic arterial pressure (SAP) are decreased, leading to reduced LV afterload, LV wall stress, MVO2 and LV preload [37]. IABP’s impact depends on balloon’s volume, aortic wall elasticity and systemic arterial resistance and can be implemented for up to 30 days [38]. Absolute contraindications include aortic dissection/ aneurysm and aortic valve insufficiency. In case of uncontrollable sepsis and increased hemorrhagic diathesis, IABP should be avoided. Most frequent complications are arterial injury, insertion-site infection and thromboembolism [39].

Several clinical trials pertaining to IABP in CS discourage its implementation at SCAI stages C-E [40–42]. In 2012, the randomized IABP SHOCK II trial recruited 598 AMICS patients prior to revascularization therapy, 300 of whom were supported with IABP. The results proved no difference in all-cause 30-day mortality among groups (RR with IABP, 0.96; 95% CI 0.79 to 1.17; *P* = 0.69) [43], altering the relevant ESC guidelines [class ΙΙb, level of evidence C in case of CS irrespective of the underlying cause and class III, level of evidence B in AMICS (not recommended)] [44]. IABP is particularly useful in SCAI stage C in CS of non-ischemic origin. Such examples are patients suffering from acute decompensation of chronic heart failure due to dilated cardiomyopathy as IABP augments stroke volume, patients with severe mitral regurgitation or increased systemic vascular resistance. Moreover, IABP can be implemented as bridge-to-transplantation or bridge-to-recovery, averting CS deterioration and implantation of advanced devices with greater complications [45–47]. Despite the IIb recommendation, IABP is often used as a first-line option in CS regardless of origin due to its substantially lower cost and simplicity in use.

## Comparison of MCS, escalation, de-escalation, and anticoagulation strategies

**Fig. 1:**
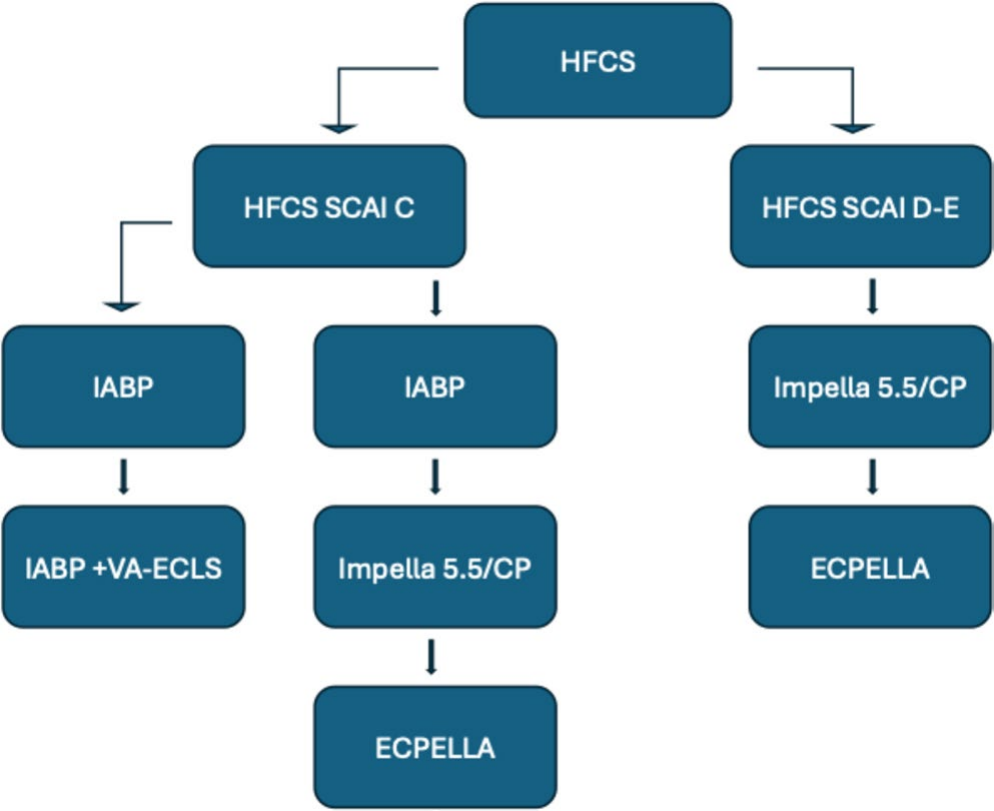
MCS selection and escalation algorithm in HFCS. ECPELLA, ECLS+Impella; HFCS, Heart failure (related) cardiogenic shock; IABP, Intra-aortic balloon pump; MCS, Mechanical circulatory support; SCAI, Society for cardiovascular angiography and interventions; VA-ECLS, Venoarterial extracorporeal life support

**Fig. 2:**
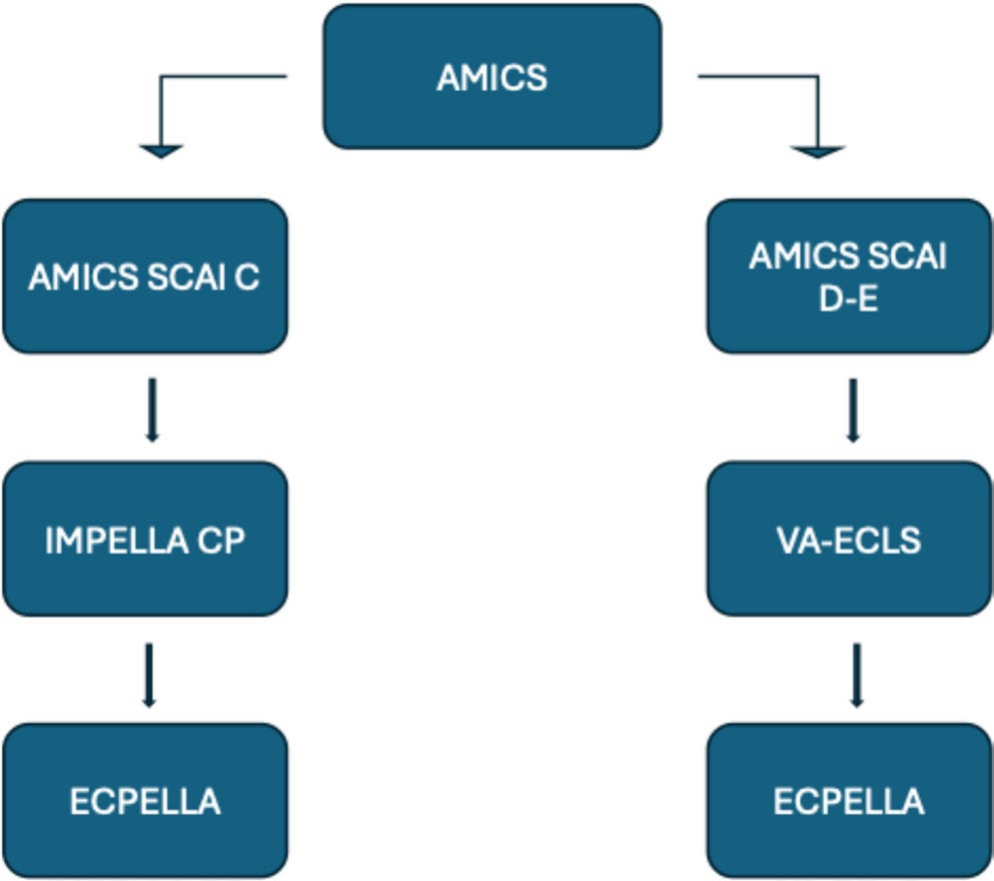
MCS selection and escalation algorithm in AMICS. AMICS, Acute myocardial infarction (related) cardiogenic shock; ECPELLA, ECLS+Impella; MCS, Mechanical circulatory support; SCAI, Society for cardiovascular angiography and interventions; VA-ECLS, Venoarterial extracorporeal life support

**Fig. 3:**
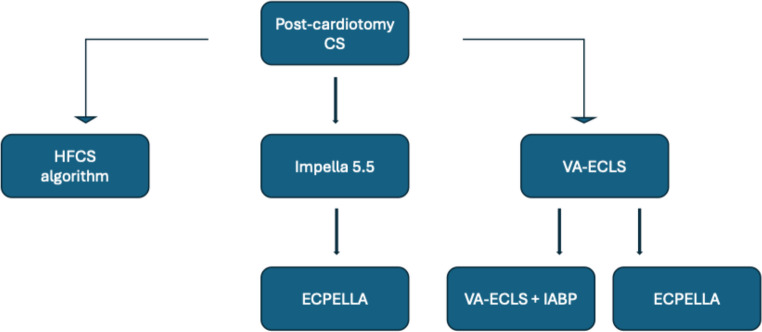
MCS selection and escalation algorithm in post-cardiotomy CS. CS, Cardiogenic shock; ECPELLA, ECLS+Impella; HFCS, Heart failure (related) cardiogenic shock; IABP, Intra-aortic balloon pump; MCS, Mechanical circulatory support; VA-ECLS, Venoarterial extracorporeal life support

An overview of available MCS options in CS is presented in Table 1. Regional protocols, familiarity with a certain device or concomitant costs influence the selection process. Our suggested algorithm presents Impella as first-line MCS. IABP is an acceptable alternative in non-AMI CS SCAI stage C. VA-ECLS is indicated in case of previous device failure or in SCAI stage D-E patients (Figs. 1, 2 and 3). Major trials and meta-analyses conducted up-to-date, show no significant difference in 30-day, 6-month and 5-year all-cause mortality among patients randomized to Impella, IABP or VA-ECLS. Complications are more frequent in Impella or VA-ECLS groups (Online Resource 8) [48–52]. Interestingly, IABP requires the simplest implantation and operation techniques. ECLS is implanted faster and requires neither transportation to operation theater nor surgical incisions, contrary to Impella 5.5, proposing a significant advantage in rapid hemodynamic deterioration. Finally, Impella CP is less invasive than VA-ECLS and preferable to IABP in AMICS.

**Table 1:** Comparison of the available MCS devices in CS

Device	Indications	Contraindications	Advantages	Disadvantages
IABP	CS, HRPCI, weaning from bypass/MCS	Aortic or femoral dissection/ aneurism ΑοV insufficiency	Easy insertion and removal, low cost	Low effectiveness, especially in AMICS and in SCAI stages C-E
IMPELLA^®^	CS, HRPCI, LV unloading during VA-ECLS	Anatomical abnormalities averting proper positioning, Valvulopathy of ΑοV/ TV/PV, RHF, shunts	Easy implantation, effectiveness, mobilization	Lower flows than VA-ECLS (transcutaneous IMPELLA), shorter duration, hemolysis, higher costs
VA-ECLS	Cardiorespiratory failure, need for BiVAD, MCS escalation, SCAI stages D-E	Noncardiac/ irreversible organ failure, patients unsuitable for MCS/ cardiac transplantation	Higher flows, prolonged support, concomitant hemodialysis if needed	Extremely invasive, frequent complications, LV loading, requires high expertise

*AMICS* aortic valve, *BiVAD* biventricular assist device, *CS *cardiac shock, *HRPCI* high risk percutaneous coronary intervention, *IABP* intra-aortic balloon pump, *LV* left vetricle, *MCS* mechanical circulatory support, *PV* pulmonary valve, *RHF* right heart failure, *SCAI* society for cardiovascular angiography and interventions, *TV* tricuspid valve, *VA-ECLS* venoarterial extracorporeal life support

The ideal antithrombotic regimen remains elusive. In CS patients gastrointestinal motility and hepatic function are reduced, resulting in decreased P2Y12 inhibitor absorption. Moreover, alterations during MCS regarding core temperature and microvascular physiology lead to platelet dysfunction. Furthermore, the implanted cannulae increase thrombotic risk, given the activation of inflammatory cascade, as well as hemorrhagic risk due to vascular injury. Consequently, intravenous administration of unfractionated heparin represents first-line treatment. Bivalirudin or argatroban are alternative options. Low-molecular-weight heparin and direct oral anticoagulants should be avoided, given their longer half-life and renal clearance. Unfractionated heparin and clopidogrel can be administered in case of AMICS post-PCI, while addition of aspirin depends on hemorrhagic risk [45].

Post MCS initiation, circulatory parameters should be re-evaluated every 6–12 h with necessary bedside interventions (Online Resource 9). MCS should be adequate to allow withdrawal of inotropic support. Indicators favoring escalation are need for concomitant inotropic-vasoactive support for more than 48 h, vasoactive-inotropic score > 20 points, persistently high LV filling pressures, pulmonary congestion, metabolic disturbances or end-organ damage. Escalation algorithms for each type of isolated LV CS are presented in Figs. 1, 2 and 3.

**Fig. 4:**
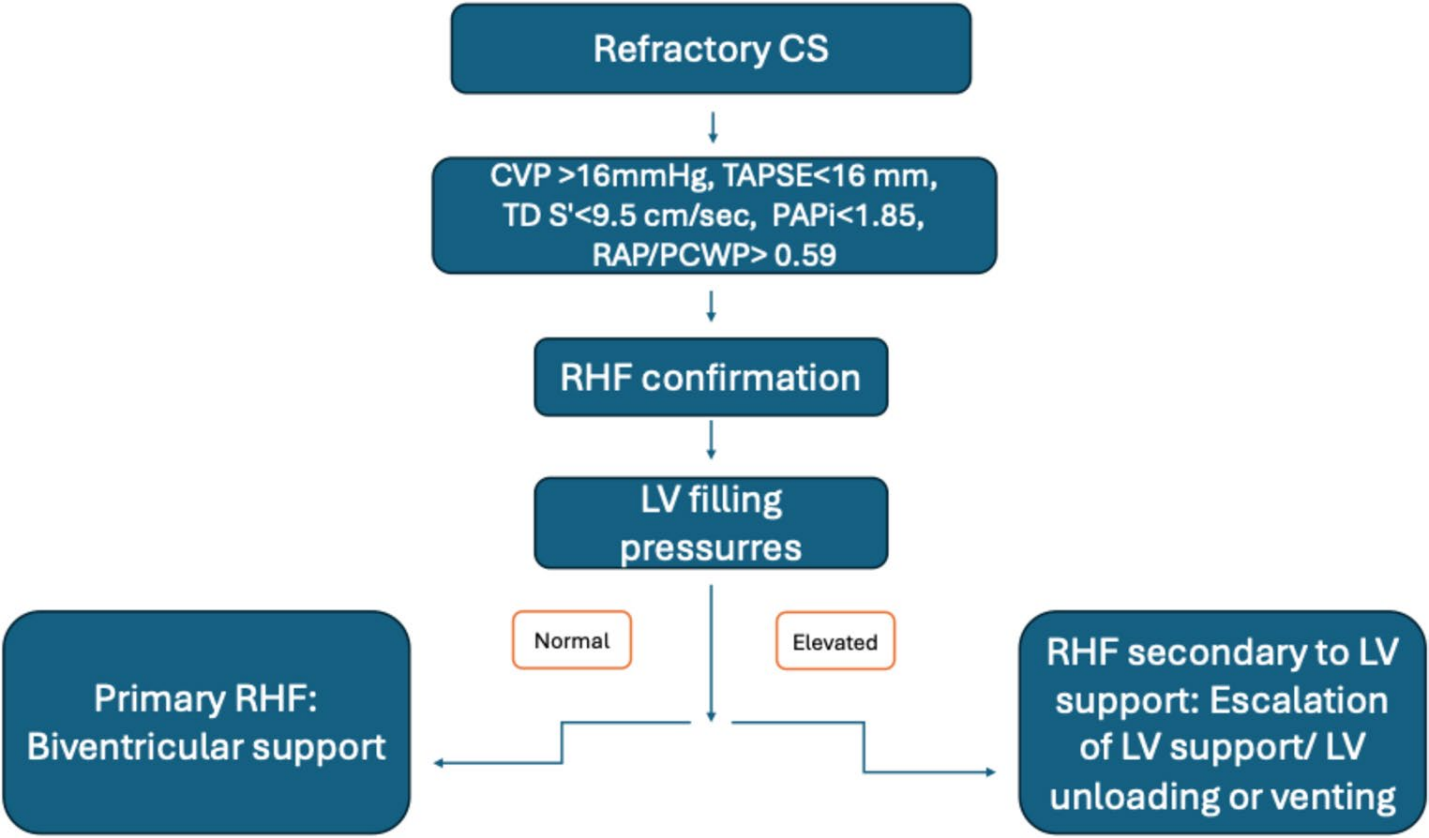
Right heart failure evolution in CS patients under MCS. CS, Cardiogenic Shock; CVP, Central venous pressure; LV, Left ventricular; MCS, Mechanical circulatory support; PAPi, Pulmonary arterial pulsatility index; PCWP, Pulmonary capillary wedge pressure; RAP, Right atrial pressure; RHF, Right heart failure; TAPSE, Tricuspid annular plane systolic excursion; TD S’, Tissue doppler systolic wave prime (velocity)

An adverse prognostic feature is evolution of RHF while on MCS, which should be suspected in patients with refractory hemodynamic instability despite optimal circulatory support. In such cases, after the exclusion of pulmonary disease, echocardiographic and hemodynamic evaluation of the RV should be performed. If LV filling pressures are elevated, then RHF has evolved secondary to LV failure and requires escalation of LV support. On the contrary, LV filling pressures are normal in primary RHF and additional RV support with Impella RP/RP-Flex or VA-ECLS and concomitant LV unloading is required (Fig. 4) [53].

According to ESC 2021 guidelines, prerequisites for MCS de-escalation include hemodynamic stability without inotropic support, low filling pressures, restored end-organ function and successful treatment of the primary pathophysiology. MCS flow is then reduced by half every 2–4 h under constant re-evaluation (Online Resources 9, 10). If attempts for MCS withdrawal are futile, decisions regarding heart transplantation, durable LVAD implantation or palliative care should be made [44].

## Future perspectives

Despite the perceived progress in temporary MCS in CS major morbidity and mortality remain unaltered. Effective implementation of a device requires expertise of health care providers in high-volume units. Thus, development of regional networks facilitating safe transportation to tertiary shock centers is crucial. Prerequisites for successful circulatory support are timely initiation (door-to-initial treatment < 30 min), continuous assessment and application of appropriate prognostication scores (SCAI, ORBI, CardShock, SAVE, IABP-SHOCK II), enabling prompt escalation. Universal time windows have not yet been defined due to great phenotypical heterogeneity and idiosyncratic response to treatment. Furthermore, protocols regarding transfusion and complex antithrombotic regimens, such as in patients with AMICS post-PCI or renal failure, should be established.

Future clinical trials should examine several MCS options with homogenous inclusion criteria, enabling proper comparison. A promising study expected in 2026, is the Cardiogenic Shock Working Group Registry-CSWG (NCT04682483), stratifying CS patients supported with all widely available devices with primary endpoints 30-day and 1-year mortality. Other areas needing clarifications are core temperature management, use of electrocardiogram-synchronized ECLS with pulsatile operation, the interplay of vasoactive agents with various MCS devices and modification of implantation techniques and devices’ characteristics [54]. Finally, cornerstone of successful CS management is the identification of proper populations for each MCS method and frequent reassessment with the use of prognostication scores to timely initiate, escalate or de-escalate MCS, minimizing occurrence of adverse events and averting end-organ damage.

## Limitations

The current article presents the main characteristics of temporary MCS in CS, highlighting their key features, most important clinical trials and operational aspects. It therefore does not systematically include all relevant trials pertaining to the examined topic. Moreover, only well-established MCS methods, namely Impella, IABP and VA-ECLS are discussed, as implementation of other devices is substantially limited in everyday clinical practice.

## Conclusion

Contemporary mechanical support options in CS patients with Impella, VA-ECLS or IABP “buy” time for myocardial recovery or bridge-to-advanced therapies. Prerequisites for effective implementation are proper device selection, timely initiation of MCS and cautious escalation and de-escalation strategies. Concomitant medical therapy, primarily comprising inotropic-vasoactive agents and antithrombotics, should be individualized. Finally, clinicians should comprehend thoroughly the complex CS pathophysiology and interactions between assisted and native circulation to obtain optimal clinical outcomes.

## Supplementary Information

Below is the link to the electronic supplementary material.

Supplementary Material 1 (350 KB)

## Data Availability

No datasets were generated or analysed during the current study.
