# Supplementary material for: Mechanical circulatory support in cardiogenic shock: a contemporary head-to-head comparison
Source: Heart Fail Rev. 2026 Mar 24;31(1):44. doi: 10.1007/s10741-026-10612-8 (PMC13013270; doi:10.1007/s10741-026-10612-8)
Supplement: Supplementary file 1 — Supplementary Material 1 (350 KB) [file 10741_2026_10612_MOESM1_ESM.pdf]

Heart Failure Reviews, Technology and Heart Failure Therapeutics Meeting collection

Supplementary Information to the article:

Mechanical circulatory support in cardiogenic shock: a contemporary head-to-head comparison.

Stavroula A. Siopi, Polychronis Antonitsis, Georgios T. Karapanagiotidis, Georgios Tagarakis, Christos Voucharas, Kyriakos Anastasiadis

Cardiothoracic Department, Aristotle University of Thessaloniki, School of Medicine, AHEPA University Hospital, Thessaloniki, Greece

Corresponding Author; Polychronis Antonitsis, E; antonits@auth.gr, T; 00302310994874

ORCID; Stavroula A Siopi, 0009-0003-0923-302X; Polychronis Antonitsis, 0000-0002-5451-4651; Georgios T. Karapanagiotidis, 0000-0002-2065-7646; Georgios Tagarakis, 0000-0001-6123-2663; Christos Voucharas, 0000-0002-4349-1597; Kyriakos Anastasiadis, 0000-0001-7837-3446

Online Resource 1 SCAI SHOCK classification

CS Stage	Presentation	Signs and Symptoms	Biochemical Indices	Hemodynamics	Interventions
**A (at risk)**	Medical history of MI or HF	Normal peripheral pulse, JVP, alertness, auscultation	Normal serum lactates, coagulation, and hepatorenal function	SAP>100 mmHg or baseline, CI>2.5 L/min/m^2, CVP ≤ 10 mmHg, PCWP ≤ 15 mmHg, PA saturation ≥ 65%	Observation
**B (beginning)**	Hemodynamic instability without hypoperfusion	Elevated JVP, fine crackles	Elevated BNP, first-degree renal de-compensation	SAP<90 mmHg, MAP<60 mmHg, or fall>30 mmHg from baseline, HR>100 bpm	Volume substitution or redistribution
**C (classic)**	Evident hypoperfusion	Disorientated, cold and clammy skin, coarse crackles, > 2s capillary refill time, urine output < 30 mL/h	Serum creatinine >1.5×baseline or > 0.3 mg/dL, or GFR<50%, lactates > 2 mmol/L, elevated BNP	CI < 2.2 L/min/m^2, PCWP>15 mmHg	Pharmacologic and mechanical support with minor modifications
**D (decline)**	Inadequate clinical response	Declining	Declining	Declining	Addition of vasoactive agents/ MCS escalation
**E (extremis)**	Collapse/ ongoing CPR	Loss of consciousness	Lactates ≥ 8 mmol/L, pH < 7.2, BD>10 mEq/L	Declining	Refractory shock

BD, Base Deficit; BNP, B-type Natriuretic Peptide; CI, Cardiac Index; CPR, CardioPulmonary Resuscitation; CS, Cardiogenic Shock; CVP, Central Venous Pressure; GFR, Glomerular Filtration Rate; HF, Heart Failure; HR, Heart Rate; JVP, Jugular Venous Pressure; MAP, Mean Arterial Pressure; MCS, Mechanical Circulatory Support; MI, Myocardial Infarction; PA, Pulmonary Artery; PCWP, Pulmonary Capillary Wedge Pressure; SCAI, Society for Cardiovascular Angiography and Interventions; SAP, Systolic Arterial Pressure. Reproduced from [2]

Online Resource 2 Comparison of technical characteristics of Impella types

**Device**	**Impella CP**	**Impella 5.5**	**Impella RP**	**Impella RP Flex**
**Indication**	CS, HRPCI	CS	RHF	RHF
**Introducer's diameter**	14 Fr	23 Fr	23 Fr	23 Fr
**Pump's diameter**	14 Fr	19 Fr	22 Fr	22 Fr
**Implantation techique**	T/C,<br>fem. / axil. art.	Surgical, axil. art. or directly in asc. aorta.	T/C,<br>fem. v.	T/C,<br>int. jug. v./ fem. v.
**Maximal mean flow (L/min)**	3.7	5.5	4.4	>4
**Maximal support duration**	HRPCI ≤6h<br>CS<5 days	5 days	14 days	14 days
**SmartAssist technology**	Yes	Yes	Yes	Yes

Impella CP and 5.5 are equipped with SmartAssist technology, which confirms the proper pump positioning and enables the hemodynamic monitoring of left ventricular pressure, end diastolic pressure, cardiac output, cardiac power output in left cardiac support, as well as pulmonary pressures, pulmonary artery pulsatility index and central venous pressure in right cardiac support. art, Artery; axil, Axillary; asc, Ascending; CS, Cardiogenic Shock; fem, Femoral; Fr, French Gauge; HRPCI, High Risk Percutaneous Coronary Intervention; int. jug. Internal Jugular; RHF, Right Heart Failure; T/C, transcutaneous; v, Vein

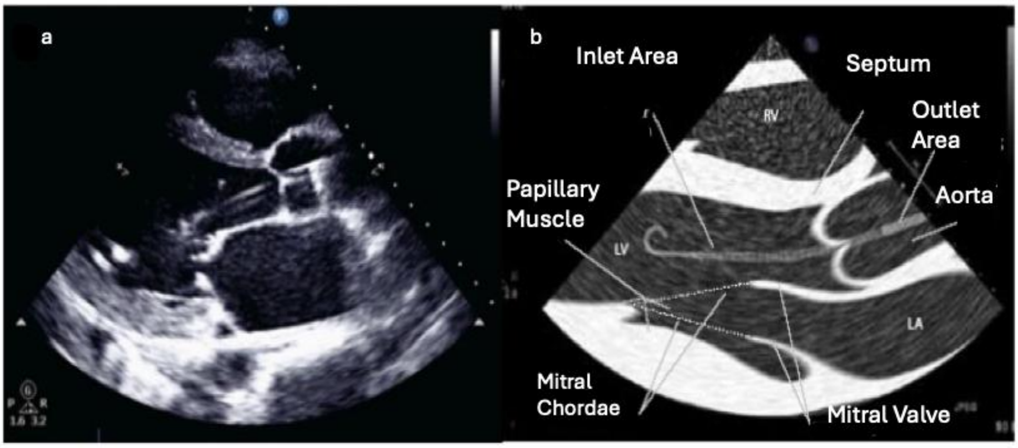

Online Resource 3 Echocardiographic evaluation of Impella positioning (**a**) Echocardiographic image **(b)** Schematic depiction. The inlet area is located in LV and the outlet area in ascending aorta.
LA, Left Atrium; LV, Left Ventricle; RV, Right Ventricle. Reused with permission from [6]

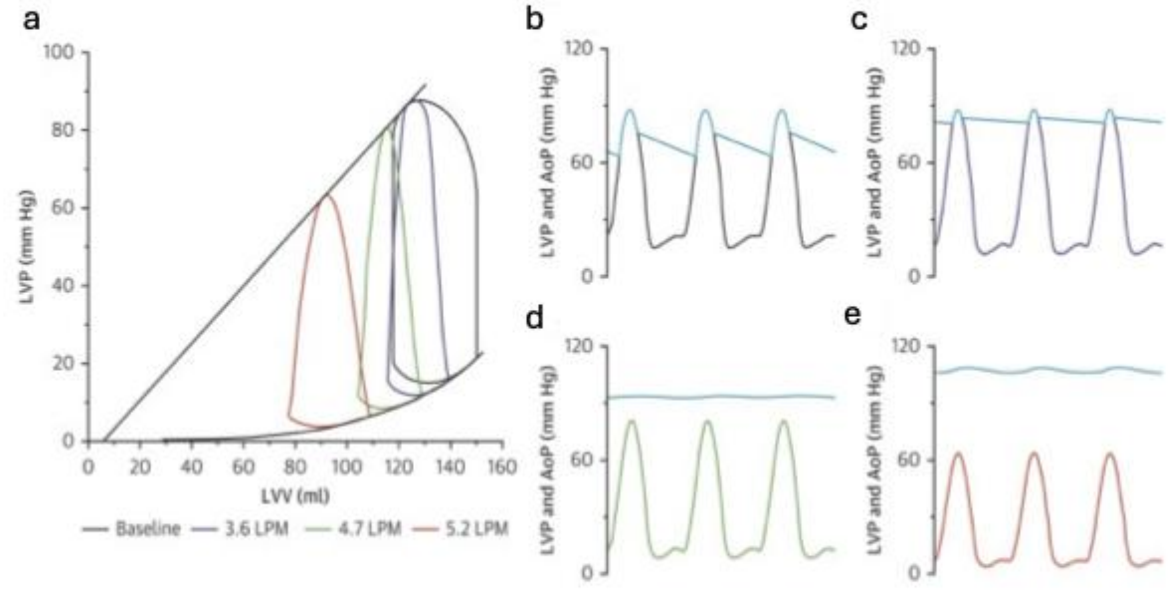

Online Resource 4 PV-Loop changes under left-sided Impella support **(a)** In case of cardiogenic shock, end-diastolic pressure volume relationship is increased, and pressure volume loop is transported rightwards. Implementation of Impella reduces end-diastolic pressure and volume, pulmonary capillary wedge pressure and right ventricular afterload, and increases cardiac output, cardiac power output, and mean arterial pressure. Isovolumic contraction and relaxation phases are eliminated, therefore resulting in a triangular pressure volume loop, transported leftwards. **(b-e)** Increase in Impella flow progressively leads to left ventricular pressure and aortic pressure uncoupling.
AoP, Aortic Pressure; LPM, Liters Per Minute; LVP, Left Ventricular Pressure; LVV, Left Ventricular Volume. Reused with permission from [11]

Online Resource 5 Impella major clinical trials

Trial	Year	Population	Intervention Group/ Group A	Control Group/ Group B	Primary Outcome
**DANGER SHOCK [14]**	2024	AMICS patients (n=355)	Impella support (n=179)	OMT (n=176)	6-month all-cause mortality was lower in Impella group (45.8% vs 58.5%, HR, 0.74; 95%, CI, 0.55 to 0.99; P=0.04)
**RECOVER RIGHT [15]**	2015	Patients with refractory RHF (n=30), either post LVAD implantation (n=18) or with MI/ post cardiotomy shock (n=12)	Implantation of Impella RP in RHF patients (n=29)	RHF patients prior to right cardiac MCS (n=30)	Impella RP improved hemodynamic indices [C.I. from 1.8 ± 0.2 to 3.3 ± 0.23 l/min/m^2 ($p < 0.001$), CVP from 19.2 ± 4 to 12.6 ± 1 mmHg ($p < 0.001$)]. 30-day survival was 73.3%
**USpella [16]**	2013	AMICS patients undergoing PCI supported with Impella 2.5 (n=154)	Group A: Impella pre-PCI (n=63)	Group B: Impella post-PCI (n=91)	Pre-PCI group revealed higher survival rates (65.1% vs.40.7%, P = 0.003) when compared to post-PCI group
**RECOVER I [17]**	2013	Post-cardiotomy CS after cardiopulmonary bypass (n=16)	Implantation of Impella 5.0 in post-cardiotomy CS patients (n=16)	Post-cardiotomy CS patients prior to MCS (n=16)	Impella 5.0 resulted in improved hemodynamics, C.I. 1.65 vs 2.7 l/min/m^2 ($P = .0001$); MAP= 71.4 vs 83.1 mm Hg ($P= .01$); PADP= 28.0 vs 19.8 mm Hg ($P < .0001$). 30-day, 3-month, and 1-year survival was 94%, 81%, and 75%, respectively

AMICS, Acute Myocardial Infarction (related) Cardiogenic Shock; CI, Confidence Interval/Cardiac Index; CS, Cardiogenic Shock; CVP, Central Venous Pressure; HR, Hazard Ratio; LVAD, Left Ventricular Assist Device; MAP, Mean Arterial Pressure; MCS, Mechanical Circulatory Support; MI, Myocardial Infarction; OMT, Optimal Medical Treatment; PADP, Pulmonary Artery Diastolic Pressure; PCI, Percutaneous Coronary Intervention; RHF, Right Heart Failure

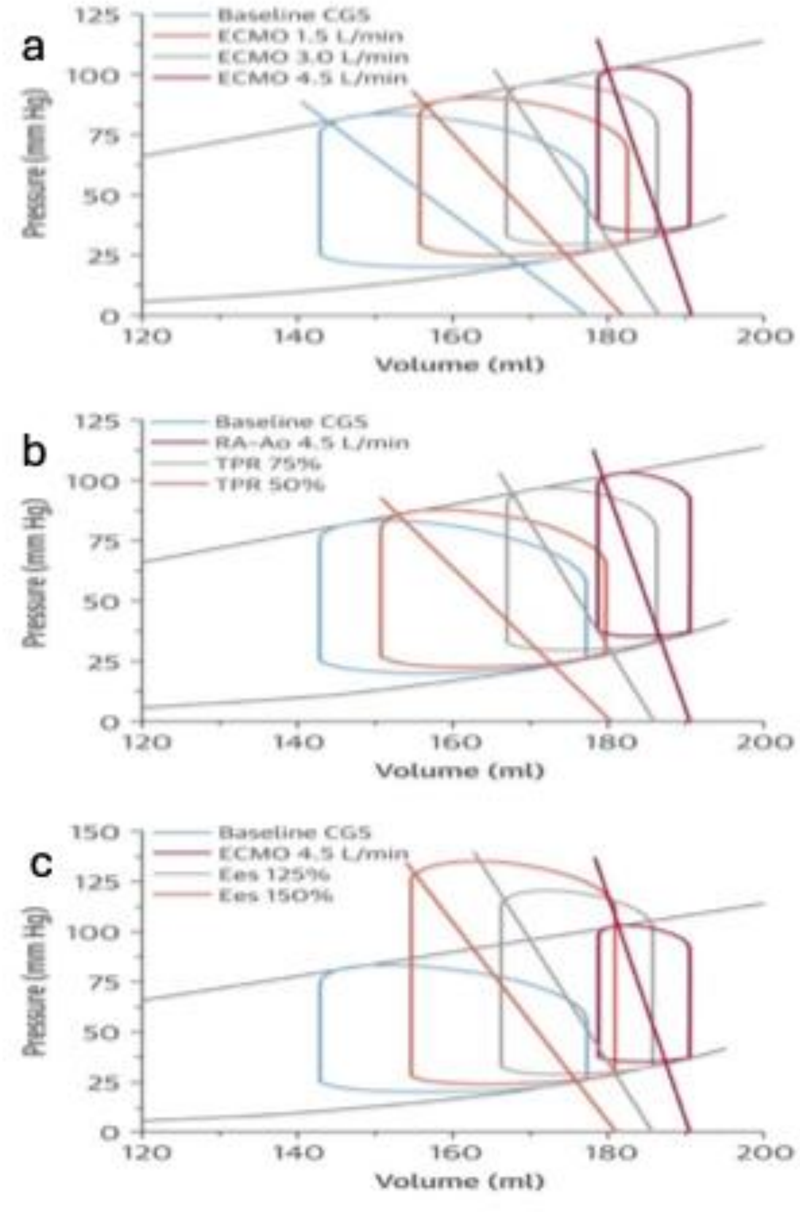

Online Resource 6 Hemodynamic effects of VA-ECLS (VA-ECMO) in native circulation a. VA-ECLS increases LV end-diastolic pressure (EDP), pressure-volume area and effective arterial elastance (Ees), while it decreases LV stroke volume (LVSV). b, c. Increase in EDP is counteracted by decrease in total peripheral resistance (TPR) or Ees.

CGS, Cardiogenic Shock; ECMO, ExtraCorporeal Membrane Oxygenation; Ees, Effective Arterial Elastance; RA-Ao, Right Atrium to Aorta; TPR, Total Peripheral Resistance. Reused with permission from [20]

Online Resource 7 Major VA-ECLS clinical trials

Trial	Year	Population	Intervention Group/ Group A	Control Group/ Group B	Primary Outcome
**ECLS SHOCK [34]**	2023	AMICS patients with planned revascularization (n=417)	VA-ECLS (n=209)	OMT (n=208)	No significant difference in 30-day, all-cause mortality (RR, 0.98; 95% CI, 0.80 to 1.19; p = 0.81)
**EURO SHOCK [35]**	2023	AMICS patients post-PCI (n=35)	VA-ECLS (n=17)	OMT (n=18)	30-day all-cause mortality was lower in VA-ECLS group (43.8% vs 61.1%, HR 0.56, 95% CI: 0.21-1.45; p=0.22)
**ECMO CS [36]**	2023	Patients in rapidly deteriorating or severe CS (n=117)	VA-ECLS (n=58)	Initial conservative treatment (n=59)	All-cause mortality, resuscitated circulatory arrest, and additional MCS at 30 days not statistically different between two groups (HR, 0.72 [95% CI, 0.46–1.12]; p=0.21)

AMICS, Acute Myocardial Infarction (related) Cardiogenic Shock; CI, Confidence Interval; CS, Cardiogenic Shock; ECMO, Extracorporeal Life Support; HR, Hazard Ratio; MCS, Mechanical Circulatory Support; OMT, Optimal Medical Treatment; PCI, Percutaneous Coronary Intervention; RR, Relative Risk; VA-ECLS, VenoArterial Extracorporeal Life Support

Online Resource 8 Major trials comparing IABP to Impella to VA-ECLS

Trial	Year	Population	Intervention Group/Group A	Control Group/Group B	Primary Outcome
**ISAR-SHOCK [48]**	2008	AMICS patients (n=25)	IABP support (n=13)	Impella LP support (n=12)	30’ post implantation CI more efficiently increased in Impella group (change 0.49 L/min/m^2, p=0.02) than in IABP group (change 0.11 L/min/m^2) (p = 0.02 for change in CI between groups). 30-day all-cause mortality was 46% in both groups
**IMPRESS [49]**	2017	AMICS patients (n=48)	Impella CP support (n=24)	IABP support (n=24)	30-day and 6-month all-cause mortality similar between groups (50% and 46%, respectively; HR with Impella: 0.96; 95% CI: 0.42 to 2.18; p = 0.92 at 30 days and 50% HR: 1.04; 95% CI: 0.47 to 2.32; p = 0.923 at 6 months)
**Lemor et al [50]**	2020	AMICS patients undergoing PCI (n=6290)	Impella support (n=5730)	VA-ECLS (n=560)	Impella associated with lower mortality rates (26.7% vs 43.3%, OR: 2.10, p = 0.021), ARDS, and vascular complications
**Karami et al [51]**	2020	AMICS patients (n=128)	Impella support (n=90)	VA-ECLS (n=38)	30-day all-cause mortality similar among groups (53% vs. 49%, *P*=0.30)

AMICS, Acute Myocardial Infarction (related) Cardiogenic Shock; ARDS, Acute Respiratory Distress Syndrome; CI, Cardiac Index/Confidence Interval; HR, Hazard Ratio; IABP, IntraAortic Balloon Pump; OR, Odds Ratio; PCI, Percutaneous Coronary Intervention; VA-ECLS, VenoArterial ExtraCorporeal Life Support

Online Resource 9 Parameters of cardiac function assessed during MCS

Parameters	Variables
**Echocardiography**	Ventricular size, LVEF, LV velocity time integral, pre-ejection and total ejection time, MAPSE/TAPSE, tissue doppler velocity, strain/strain rate, valvular lesions
**Hemodynamic, respiratory and metabolic indices**	Pulse-oximetry, invasive systemic and pulmonic pressure measurements, metabolic status
**Non-invasive monitoring**	Near infrared spectroscopy, optical nerve shear diameter
**Coagulation and cardiac biomarkers**	Activated clotting time, aPTT, anti-Xa levels, BNP, NT-pro-BNP, hs-TnI

aPTT, activated Partial Thromboplastin Time; BNP, B-type Natriuretic Peptide; hs-TnI, high-sensitivity Troponin I; LV, Left Ventricle; LVEF, Left Ventricular Ejection Fraction; MAPSE, Mitral Annular Plane Systolic Excursion; MCS, Mechanical Circulatory Support; NT-pro-BNP, N Terminal Prohormone (of) Brain Natriuretic Peptide; TAPSE, Tricuspid Annular Plane Systolic Excursion

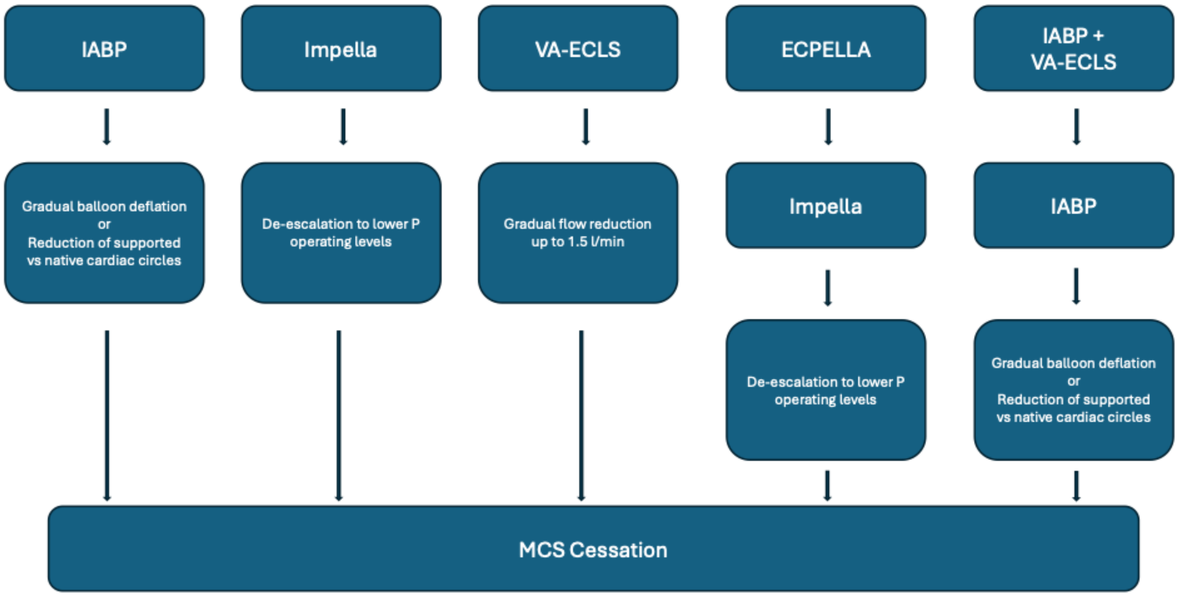

Online Resource 10 De-escalation of MCS algorithm
ECPELLA, ECLS+Impella; IABP, Intra-Aortic Balloon Pump; MCS, Mechanical Circulatory Support; VA-ECLS, VenoArterial ExtraCorporeal Life Support
